# Text Message Behavioral Intervention for Teens on Eating, Physical Activity and Social Wellbeing (TEXTBITES): Protocol for a Randomized Controlled Trial

**DOI:** 10.2196/16481

**Published:** 2020-02-18

**Authors:** Stephanie R Partridge, Rebecca Raeside, Anna C Singleton, Karice Hyun, Zoe Latham, Alicia Grunseit, Katharine Steinbeck, Clara Chow, Julie Redfern

**Affiliations:** 1 Westmead Applied Research Centre Faculty of Medicine and Health University of Sydney Sydney Australia; 2 Charles Perkins Centre, Prevention Research Collaboration Sydney School of Public Health University of Sydney Sydney Australia; 3 Department of Weight Management The Children’s Hospital Westmead Sydney Australia; 4 Discipline of Child and Adolescent Health Faculty of Medicine University of Sydney Sydney Australia; 5 The George Institute for Global Health University of New South Wales Sydney Australia

**Keywords:** obesity, adolescents, nutrition, physical activity, text message, randomized controlled trial, mHealth

## Abstract

**Background:**

Obesity is among the most significant health challenges facing today’s adolescents. Weight gain during adolescence is related to cardiovascular disease, type 2 diabetes, and some cancers in later life. Presently, adolescents living in Australia have limited access to age-appropriate obesity prevention services.

**Objective:**

This study aims to investigate whether a two-way text message program, with optional telephone health counseling, improves body mass index (BMI) *z* score and lifestyle outcomes in adolescents who are overweight.

**Methods:**

This study will be a single-blind randomized controlled trial (N=150) comparing a two-way text message intervention, with optional telephone health counseling, to usual care in adolescents (13-18 years old, inclusive) who are overweight (recruited from a pediatric weight management clinic and the broader community in Sydney, Australia). The intervention group will receive a six-month text message program, which consists of two-way, semipersonalized, lifestyle-focused text messages (four messages/week) in addition to usual care. The control group will be assigned to receive usual care. The study also includes a follow-up at 12-months. The primary outcome is a change in BMI *z* score at six months. Secondary outcomes are changes in waist-to-height ratio, diet, physical and sedentary activity levels, sleep quality, quality of life, self-esteem, self-efficacy, social support, and eating disorder and depression symptoms. Also, we will examine acceptability, utility, and engagement with the program through a study-specific process evaluation questionnaire, semi-structured telephone interviews, and an analysis of health counselor communication logs. The analyses will be performed by the intention-to-treat principle to assess differences between intervention and control groups.

**Results:**

The study opened for recruitment in December 2019. Data collection is expected to be completed by December 2021, and the results for the primary outcome are expected to be published in early 2022.

**Conclusions:**

This study will test the effectiveness of an interactive two-way text message program compared to usual care in improving BMI *z* score and lifestyle outcomes in adolescents with overweight. This interactive, innovative, and scalable project also aims to inform future practice and community initiatives to promote obesity prevention behaviors for adolescents.

**Trial Registration:**

Australia New Zealand Clinical Trials Registry (ANZCTR) ACTRN12619000389101; https://www.anzctr.org.au/Trial/Registration/TrialReview.aspx?id=377158&amp;isReview=true

**International Registered Report Identifier (IRRID):**

DERR1-10.2196/16481

## Introduction

The global prevalence of obese and overweight adolescents has significantly increased over the last five decades, with 18% of the global population of children and adolescents being overweight or obesity in 2016 [1]. No developed countries have successfully halted the continuing upward trend in the prevalence of obesity and being overweight since 1980 [1]. For example, in 2017-2018, 25% of adolescents aged 12-17 years old and living in Australia, were overweight or obese [2]. Among older adolescents (16-17 years old) residing in Australia, there has been a 57% increase in the incidence of obesity in the last three years [2]. In the United States of America, 20.6% of adolescents aged 12-19 years old had obesity in 2013-2014 [3].

Preventing obesity during adolescence is important because adolescence is a life stage when risk factors and lifestyles are established [4,5]. Once obesity is established, weight loss and weight maintained after weight loss are difficult to achieve [6]. Consequently, gaining excess weight during adolescence is likely to lead to being overweight and obese in adulthood [7]. Weight gain during adolescence is associated with higher risk and earlier onset of cardiovascular disease [8], type 2 diabetes [9], and some cancers [10]. Moreover, adolescent obesity has adverse psychosocial outcomes, including weight stigma [11] and reduced quality of life and self-esteem [12]. Long term weight regulation is related to diet and physical behaviors that are adopted during adolescence and track throughout life [4]. Therefore, to halt the rise in the prevalence of overweight and obese adolescents, scalable, low-cost, and engaging strategies are needed.

Several lifestyle risk factors have been associated with excess weight gain during adolescence, and adolescents that are living in developed countries are not achieving national recommendations. Firstly, consuming the recommended intake of fruits and vegetables may prevent unhealthy weight gain [13], yet only 4% of adolescents living in Australia, aged 12-17 years old, meet national guidelines for fruit and vegetable intake [2]. Secondly, adolescents remain the highest consumers of discretionary foods and sugar-sweetened beverages, despite the evidence that consumption increases the risk of obesity and being overweight [14,15]. In Australia and the United States, over 60% of adolescents drink a sugar-sweetened beverage daily, adding 143 kilocalories per day. Moreover, discretionary foods account for a significant portion of adolescents’ total energy intake (>40% in Australian adolescents) [16]. Finally, short sleep duration [17], insufficient physical activity levels, and increased screen time are associated with an increased risk of obesity [18,19]. Both physical activity and sedentary screen-based behavior guidelines are met by less than 4% of adolescents aged 13-17 years old [20], and night-time technology use can have harmful effects on adolescent sleep duration [21].

Despite the apparent risk for weight gain during adolescence, evidence to inform effective interventions for this population is lacking. A 2019 Cochrane review of 31 randomized controlled trials (RCTs) testing the effectiveness of a range of interventions, which included diet or physical activity components or both, designed to prevent obesity in adolescents found limited effective interventions for adolescents aged 13-18 years old [22]. There has been more investment in childhood obesity prevention research, with over 85 RCTs conducted in children aged 6-12 years old. Moreover, the diet and physical activity strategies delivered to adolescents in the studies did not reduce their body mass index (BMI) *z* score, and there has been limited investigation of digital intervention modalities. Current attrition rates for traditional, in-person obesity prevention and management interventions in adolescents remain highly variable, with 27% to 73% of participants dropping out of interventions for reasons including the intervention not meeting the adolescent’s expectations [23,24]. Digital technology has been identified as an engaging intervention modality for overweight and obese adolescents [25]. Thus, mobile phone interventions hold promise for delivering interventions that are scalable, low-cost, and engaging.

Currently, over 90% of adolescents living in Australia and the United States own a mobile phone [26,27], and text messaging remains a primary means of communication between adolescents [27]. There is a small body of evidence which indicates one-way text message interventions can promote weight loss in adults [28]. Emerging research has shown that text messages are a feasible and acceptable form of communication in interventions for adolescents with obesity [29-32]. However, there is limited high-quality evidence for the role of two-way text messages to improve obesity prevention behaviors in adolescents who are overweight. Therefore, the primary aim of this study is to test the effectiveness of a two-way, semipersonalized text message program, with optional health counseling, compared to usual care for improving adolescents’ BMI *z* score. This study also aims to determine if the text message program can improve lifestyle outcomes. The study will also examine acceptability, utility, and engagement with the program.

## Methods

### Study Design

**Figure 1:**
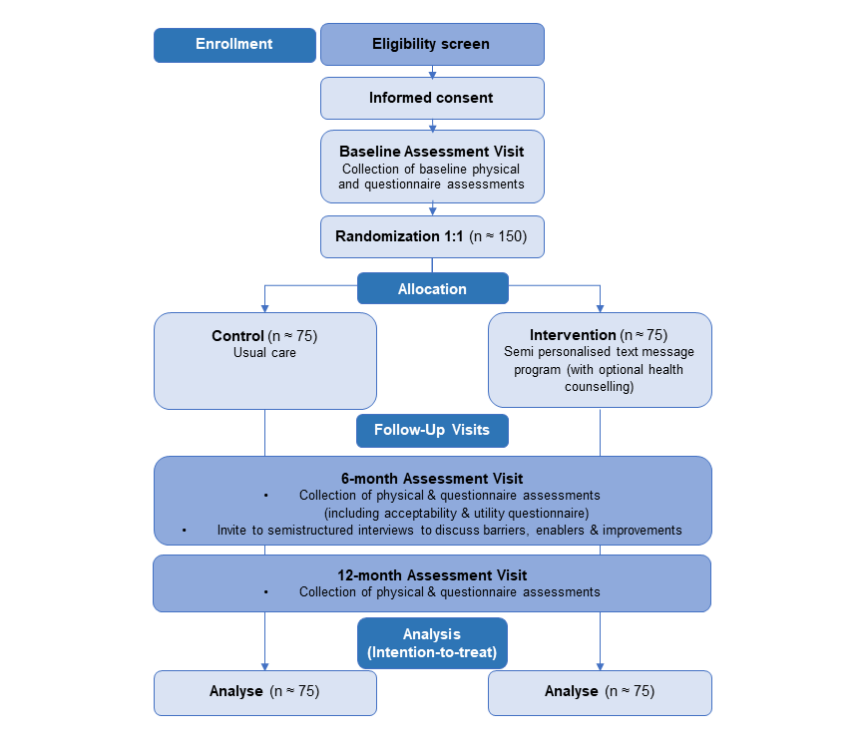
Flow chart of study design. Adolescent participants 13-18 years, with overweight are considered for inclusion in the study and randomization is single blind, with allocation determined and initiated by the computerized program.

The TEXTBITES (TEXT message Behavioral Intervention for Teens on Eating, physical activity, and Social wellbeing) study is a single-blind, multi-center, randomized controlled trial, which delivers a six-month obesity prevention program via text messages to adolescents who are overweight. The study also includes a twelve-month follow-up (Figure 1). The sites include public hospitals and a pediatric weight management clinic at a public children’s hospital in Sydney, Australia. Participants will be recruited via referral from the pediatric weight management clinic, and from the local community via print and digital advertisements at community centers, high schools, and tertiary education providers. A log of all recruitment strategies utilized will be kept. The target sample size is 150 adolescents aged 13-18 years old inclusive, who are overweight. Participants will be randomly allocated to either a control or intervention group. The control group will be assigned to receive usual care. For this study, usual care is defined as accessing available health services and information for adolescent obesity prevention. The intervention group will be allocated to receive the six-month, two-way text message program for obesity prevention that provides practical tips and information, motivation, and support for lifestyle modification for healthy eating, physical activity, and mental wellbeing behaviors, in addition to usual care. Trained research personnel blinded to the group allocation will conduct assessments at baseline, six-, and twelve-months during a face-to-face interview.

### Randomization and Blinding

After obtaining written informed consent from adolescents and their parents or guardians (if <18 years old) and completing the baseline assessment, the research assistant will enter the data into a secure web-based database. Then randomization will occur via a centralized, computerized randomization program in a uniform 1:1 allocation ratio (control: intervention). Randomization is based on a permuted randomized block design, where the strata are gender, age, and recruitment site. The randomized block method contained block sizes of 2 and 4 in a ratio of 2:1 to minimize imbalance in small strata groups. A randomization list was generated by an independent statistician using RandomiseR package [33] within the R computing environment (The R Foundation, Vienna, Austria). For each participant, the computer system automatically produces a study identification number, which will be used on all study documents. On the following Monday, after the baseline visit, the computer system automatically sends the assigned text message program to the participant. Therefore, the researcher conducting all face-to-face assessments remains blind to the study allocation. As an additional precaution and for safety purposes, there will be an independent, unblinded researcher monitoring all incoming text messages.

A 20% (30/150) subsample of participants will be randomly assigned to wear an accelerometer for seven days to validate the self-reported physical activity questionnaire. These participants will receive a second layer of randomization, which will be nested within each treatment arm at a ratio of 1:5. A total of 15 participants with accelerometers will be in the text message intervention group, and 15 participants will be in the control group.

### Study Population

Participants will be eligible to participate if they are: (1) 13 to 18 years old inclusive; (2) are overweight, defined by the International Obesity Task Force as equivalent to an adult BMI of 25.0-29.9 kg/m^2^ [34,35]; (3) own an operational mobile phone that is capable of sending and receiving text messages; and (4) provide written informed consent themselves or provide written informed consent from their parents or guardians (if <18 years old).

Participants will be excluded if they: (1) have a diagnosis of Type 1 diabetes or Type 2 diabetes; (2) have a medical condition or psychiatric illness that would not allow the participant to give informed consent or would preclude the participant’s ability to comply with the study protocol; (3) a history of disordered eating, including being diagnosed with, or treated for, Anorexia nervosa or Anorexia athletica, Binge Eating Disorder, or Bulimia nervosa; (4) are pregnant, or are planning to become pregnant within the next 12 months; (5) are on weight loss medications or any medications known to cause weight gain; (6) are enrolled in an alternative randomized weight management program; (7) are already participating in a text message-based study; or (8) cannot speak English. The team will keep recruitment and screening logs for those people who are ineligible or decline to participate, with basic demographic information and reasons for nonparticipation.

### Control Group

The control group will receive usual care. For this study, usual care is defined as accessing available health services and information for adolescent obesity prevention, as outlined in the clinical practice guidelines for overweight and obese adults, adolescents, and children in Australia [36]. Participants in the control group will receive an initial text message welcoming them to the study. However, they will not receive the text message support program or health counseling telephone calls. The control group will also receive a text message reminder approximately six- and twelve-months after their enrolment, notifying them that they will be contacted to schedule their six- and twelve-month follow-up visits, respectively. Participants in the control group will be offered the opportunity to receive the text message support intervention (no health counseling) at the end of the study (twelve-month visit) if they wish.

### Intervention Group

#### Overview

The intervention group will receive usual care, plus a text message support program that includes a series of text messages focused on lifestyle modification for obesity prevention, as well as an opportunity to speak with an English-speaking health counselor over six months, as detailed below.

#### Message Content, Frequency, and Sequence

The text message content was co-designed with adolescents and research/health professionals and using established scientific methods [37]. The bank of 107 unique text messages are based on seven behavior change techniques with demonstrated effectiveness in adolescent obesity prevention interventions [38], and on evidence-based health information found in current national obesity prevention, nutrition, and physical activity guidelines [36,39,40]. Findings from previous text message development research with adolescents were also applied to ensure the text message style and language were appropriate and engaging for adolescents [31,32,41,42].

Each intervention participant will receive a customized and semipersonalized set of text messages sent on four random days per week, including one weekend day, and at random times (Table 1). Each unique text message will only be sent only once during the 6-months. If the participant is attending high school, the weekday text messages will only be sent before or after school hours (8:00 AM to 9:00 AM or 3:30 PM to 7:30 PM). Text messages are based on four priority areas, namely, healthy eating behaviors (n=26), physical activity behaviors (n=18), mental wellbeing (n=21), and general behaviors (n=34), and a total of 6 text messages prompt communication with the health counselor. One welcome message and one final text message are included in the bank of 107 text messages. General behavior messages are focused on the environmental impact of eating and activity, food environment, time management, and practical tips.

The text messages will be semipersonalized by using the participant's name and selecting text message content relevant to the participant's characteristics, such as age. Participants can also update their personal information (eg, mobile phone number or change in school attendance) throughout the study. Messages are sent at no cost to the participants; however, text message replies to the study team will be paid for by the participant at standard short message service (SMS) rates set by their mobile phone provider. The intervention will encourage two-way communication, with participants monitored by the health counselor on weekdays. All replies and responses will be reviewed in regular weekly team meetings.

**Table 1:** Examples of text messages sent to the intervention group.

Text message category	Example text message
Introductory	*Hi [pref_name]^a^, welcome to the TEXTBITES^b^ study. We hope you find the program fun and helpful. If you have any questions throughout the program, text us or call. We are here to support you! If you ever want to stop the messages, respond STOP to opt-out.*
Physical activity	*Need a dose of some happy hormones? Stretching can release endorphins, reduce your stress and make you feel great. The best part? You can do it anywhere, even while watching TV or YouTube. Check it out: tinyurl.com/stretchyout.*
Nutrition	*Corn isn’t just a tasty snack, it’s multi-purpose! It can be used to make fireworks, glue, paint & plastic. But let's face it, popcorn is one of the best uses, check out some recipe ideas here: tinyurl.com/airpopcorn.*
Mental wellbeing	*Your brain has 86 billion thinking cells (called “neurons”) & 80 billion or so supporting cells. Wow, that's a lot! No wonder they need a break at night to rest & recover. Make sure to give them 8-10 hours rest each day!*
General behaviors	*Want to get your homework or study done in record time? Do power bursts! Put your phone on do not disturb and power it out for 25 min. Take a 5 min break (yep, you can check your phone) and repeat until your work is done!*
Health counselor	*How is everything going, [pref_name]? Text back if you would like to chat with our health counsellor about all things food, exercise and wellbeing and we will get back to you soon.*
Final	*This is your last message from the TEXTBITES study. Thank you for being a part of the program, we couldn’t have done it without you! We will contact you shortly to arrange your 6-month follow-up interview. Thanks again from the TEXTBITES study team!*

^a^pref_name: participants preferred first name

^b^TEXTBITES: TEXT message Behavioral Intervention for Teens on Eating, physical activity, and Social wellbeing

#### Role of the Health Counselor

Once a month, over six months, intervention participants will be sent a text message encouraging them to call the university-qualified health counselor to ask questions or request additional information (Table 1, example, two-way communication messages). The health counseling calls will employ complementary theoretical approaches to the text messages, including motivational interviewing, goal setting, self-monitoring, barrier identification, and problem-solving [43,44]. The personalized health counseling calls will last 10-15 minutes and will be delivered according to a standardized protocol. The university-qualified health counselor (allied health professional) will monitor and respond to participants’ request for a call each month, either via text message or phone call, within three working days. Participants are allowed a total of six health counseling calls in total over six months. The health counseling calls will enable participants to set behavioral goals, discuss barriers and enablers to behavior change, and their overall progress. This part of the intervention is based on the evidence based TEXTMEDS (TEXT messages to improve MEDication adherence and Secondary prevention) study for secondary prevention of heart disease [45].

### Text Message Management System

The text message management system was developed in conjunction with the text message intervention to support women's physical and mental health after breast cancer treatments study and has similar processes [46]. Each week messages will be selected from the message bank by the software sequencer system that ensures that participants are receiving the correct messages (intervention vs control). Both intervention and control groups will receive a welcome text message at the beginning of the study and a concluding message at the end of the 6-months. Each text message will have a unique signature to ensure that participants know these messages are from the TEXTBITES study. All participants will be given brief training at baseline on how to read, delete, and save a text message, and how to unsubscribe if required. Training will also include safe and acceptable times to read the text messages (eg, reminding participants they must not read the text messages or use any other mobile phone functions if they are driving). All participants will also be provided with the research personnel’s contact details and will be contacted at least once during the intervention period to organize the six-month follow-up assessment and once during the follow-up period to facilitate the twelve-month follow-up assessment.

A researcher will manage a study mobile phone, and a record will be kept of any incoming messages from participants, and all out-going replies from the study health counselor throughout the study. Any analysis of these incoming text messages will be performed at the group level, except for reporting examples of individual quotes, which will be anonymized to protect the participant’s identity. Participants from either group can withdraw from the study at any time with or without giving a reason by replying “STOP” to any of the messages or contacting a member of the research team, which will activate a process of review and withdrawal from the study. If a reason for withdrawal is provided, it will be recorded.

### Data Collection and Study Outcomes

The in-person follow-up assessments will occur at the end of the six-month intervention period and the end of the six months post-intervention (twelve months from baseline). The primary outcome, secondary outcomes, and their assessments are presented in Table 2. The primary outcome is a change in BMI *z* score (units BMI is above or below average for age- and sex-specific reference values) measured using calibrated stadiometer and electronic scales. BMI is calculated as weight (kg)/[height (meters)^2^]. Bodyweight and height will be measured to the nearest 0.1 kilogram and 0.1 centimeter, respectively, at each assessment time point by the research assistant, who is blinded to participant allocation and is using a standardized protocol [47]. Waist-to-height ratio will be measured to the nearest 0.1 centimeter using a nonstretch plastic waist measurement tape, midway between the iliac crest and the lowest rib, and a calibrated stadiometer.

**Table 2:** Description of TEXTBITES study outcomes and assessments.

Outcome		Assessment
**Primary outcome**		
	BMI^a^* z* score	Units BMI is above or below average for the age- and sex-specific reference values measured using calibrated stadiometer and electronic scales [47].
**Secondary outcomes**		
	Waist-to-height ratio	The midway measurement between the iliac crest and lowest rib and height, measured using a non-stretch plastic waist tape and a calibrated stadiometer [47].
	Adherence to dietary guidelines	Short questions adapted from the New South Wales Population Health Survey [48]
	Diet quality, food choices and food patterns	ACAES^b^ Survey [49]
	Physical activity	Validated short physical activity question and study-specific sports participation questions [50]
	Sedentary activity	Modified ASAQ^c^ [51-53]
	Objective physical activity	Actigraph GT3X+ activity monitors worn for seven days [54,55]
	Sleep quality	PSQI^d^-Short [56,57]
	Quality of life	PedsQL^e^ Version 4.0 Generic Core Scales questionnaire [58]
	Self-esteem	Rosenberg Self-Esteem Scale [58]
	Self-efficacy	Short questions adapted from the Project Eat Survey II [59]
	Social support	Short questions adapted the social support and eating habits survey [60] and Social Support Scale for Physical Activity [61]
	Eating disorders	EDE-Q^f^ [62]
	Depression	CESDR-10^g^ [63]

^a^BMI: body mass index

^b^ACAES: Australian Child and Adolescent Eating Survey

^c^ASAQ: Adolescent Sedentary Activity Questionnaire

^d^PSQI: Pittsburgh Sleep Quality Index

^e^PedsQL: Pediatric Quality of Life Inventory

^f^EDE-Q: Eating Disorder Examination questionnaire

^g^CESDR-10: Centre for Epidemiological Studies Depression Scale-Revised-10

The following questionnaire-based assessments have demonstrated validity and reliability in adolescent populations and will be completed online at the in-person follow-up assessment. Diet quality, food choices, and food patterns will be measured using the Australian Child and Adolescent Eating Survey (ACAES) [49], and adherence to dietary guidelines will be measured using short questions adapted from the North-South Wales Population Health Survey [48]. Physical activity and sedentary behaviors will be measured using a validated short physical activity question, study-specific sports participation questions [50], and a modified version of the Adolescent Sedentary Activity Questionnaire (ASAQ) [51-53]. For data quality, physical activity and sedentary behaviors will also be objectively assessed in a random 20% (30/150) subsample of participants using Actigraph GT3X+ activity monitors worn for seven days [54,55]. Sleep quality, quality of life, and self-esteem will be measured using the Pittsburgh Sleep Quality Index Short (PSQI-Short) [56,57], the Pediatric Quality of Life Inventory (PedsQL) Version 4.0 Generic Core Scales questionnaire [58], and the Rosenberg Self-Esteem Scale [58], respectively. Self-efficacy will be assessed using a questionnaire adapted from Project Eat Survey II [59], and social support will be assessed using a questionnaire adapted from the social support and eating habits survey [60] and the Social Support Scale for Physical Activity [61]. Eating disorders will be measured using the Eating Disorder Examination Questionnaire (EDE-Q) [62], and depression will be measured using the Centre for Epidemiological Studies Depression Scale Revised-10 (CESDR-10) [63]. The schedule of enrolment, interventions, and assessments are presented in Table 3.

**Table 3:** TEXTBITES study schedule of enrolment, interventions, and assessments.

			Enrolment		Allocation		Postallocation			
Assessments			Before baseline (–t_1_)		Timepoint 0		Baseline (t_1_)		6-months (t_2_)	12-months (t_3_)
**Enrolment**										
	Eligibility screen	✓								
	Informed consent	✓								
	Allocation			✓						
**Interventions**										
	Intervention group					✓		✓		
	Control group					✓		✓		
**Assessments**										
	BMI^a^* z* score					✓		✓		✓
	Waist-to-height ratio					✓		✓		✓
	Adherence to dietary guidelines					✓		✓		✓
	Diet quality, food choices and food patterns					✓		✓		
	Physical activity					✓		✓		✓
	Sedentary activity					✓		✓		✓
	Objective physical activity					✓		✓		✓
	Sleep quality					✓		✓		✓
	Quality of life					✓		✓		✓
	Self-esteem					✓		✓		✓
	Self-efficacy					✓		✓		✓
	Social support					✓		✓		✓
	Eating disorders					✓		✓		✓
	Depression					✓		✓		✓

^a^BMI: body mass index

### Process Measures

The adolescents’ acceptability, utility, and engagement with the two-way semipersonalized text message intervention and interaction with a health counselor will be measured using a study-specific, process evaluation questionnaire, and semistructured telephone interviews. All intervention participants will be asked to complete the process evaluation questionnaire at their 6-month follow-up assessment. The questionnaire will include questions regarding acceptability and utility that require Likert responses from strongly agree to strongly disagree. It also includes open-ended questions about the most useful and least useful components of the program, as well as suggestions to improve the program. Measures of engagement with the program will be extracted from the text message management system, including the number of text messages sent and replies received. At the 6-month follow-up, both control and intervention participants will be asked if they accessed any other health-related information or programs regarding weight management or lifestyle behavior change. Intervention participants will be invited in person, or by telephone, to take part in a semistructured telephone interview at six-months. A minimum of 15 interviews will be conducted and will be consecutive until no new themes or categories emerge (thematic saturation). However, it is anticipated that at least 30-40 participants will be invited to take part. These interviews will be conducted by telephone and will last approximately 30 minutes. Participants will be purposively selected to ensure that a variety of views are explored and to obtain a mix of participants in terms of age and ethnicity.

### Statistical Considerations

Modest reductions in BMI *z* score (0.01-0.15) in adolescents have been associated with improvements in several cardiovascular risk factors and are considered to be clinically meaningful [64]. The population SD for this power calculation was obtained from a contemporary Australian RCT in adolescents [65]. To test for a change in BMI *z* score of 0.15 (SD 0.27) [65] in the intervention group at six-months, compared with the control group, for 80% power (type I error=5%, two-sided test), 1:1 randomization, and accounting for a conservative 30% dropout-rate based on a recent systematic literature review [66], we will require 150 participates (75 participants per group).

Baseline demographic characteristics, attrition rates, and the average number of text messages sent and received for participants will be tabulated and compared using descriptive statistics. Categorical variables will be summarized using totals numbers and percentages. Continuous variables will be summarized using mean and standard deviation for normally distributed data, or median and interquartile range for data that are not normally distributed. The baseline characteristics of completers and noncompleters will be compared to examine attrition bias, using two-tailed *t* tests for continuous variables and chi-square tests for categorical variables. Actigraph GT3X+ accelerometer data will be captured in 1-15 second epochs to capture the intermittent activity patterns of adolescents and downloaded using the ActiLife software into an excel spreadsheet. Time spent in different physical activities will be evaluated by classifying the intensity level using count thresholds specific to adolescents [54,55].

The primary analysis will include all available participant data and will be performed at the end of the study after all the data has been collected. The analysis of the primary and secondary outcomes will be conducted according to the intention-to-treat principle. Continuous outcomes will be analyzed at six- and twelve-months using analysis of covariance (ANCOVA), adjusting for baseline measurement of the outcome, gender, and recruitment site/strategy. Categorical outcomes will be analyzed at six- and twelve-months using log-binomial regression and adjusting for baseline measurement of the outcome, gender, and recruitment site/strategy. Planned subgroup analyses will investigate interactions between treatment and subgroups, including categories of age, socioeconomic status, and ethnicity, to explore trends for future studies. A significance level of 0.05 will be used. All analyses will be undertaken using SAS version 9.4 (SAS Institute Inc, Cary, North Carolina, United States).

Process evaluation data, including questionnaire data, interview data, and text message management data, will be analyzed using mixed methods. Descriptive analysis will be used to examine questionnaire data and software analytics. Interviews will be conducted by a trained qualitative interviewer, digitally recorded and transcribed. Interview data will be analyzed thematically, and coding based on emergent themes using the NVIVO Software program, version 12 (QRS International Pty Ltd, Victoria, Australia).

### Dissemination

The study findings will be disseminated via peer-reviewed publications and presentations at national and international conference meetings. Results will also be presented and communicated appropriately to relevant adolescent and consumer groups.

### Ethical Approval and Consent to Participate

Formal ethical approval for this study has been obtained from the Sydney Children’s Hospitals Network Human Research Ethics Committee (approval number HREC/18/SCHN/374). The current protocol version is Version 2.0 (March 20, 2019). Written and informed consent will be collected from all participants and their parents/guardians (if they are <18 years old). The study is sponsored by the University of Sydney and managed by staff based there. The sponsor has no role in the study design, the collection, management, analysis, and interpretation of data, the writing of the findings, or the decision to submit the findings for publication. The design and conduct of the study will be overseen by a steering committee (authors). This study will adhere to the Australian National Health and Medical Research Council ethical guidelines for human research, and the study will follow the Consolidated Standards of Reporting Trials guidelines [67].

## Results

Recruitment for the trial will start in December 2019 and is expected to run until the end of 2020. Data collection is expected to be complete by December 2021, and dissemination of trial results is planned after that. The results on the primary outcome are expected to be ready during early 2022.

## Discussion

### Primary Findings

The study will evaluate an innovative means of delivering a simple obesity prevention program to adolescents who are overweight and are at risk of obesity using an RCT. It is hypothesized that the intervention group will see improvements in primary and secondary outcomes compared to control post-intervention (six-months) and at follow-up (twelve-months). If effective, this study will inform translational research to improve BMI and lifestyle outcomes for adolescents, and to prevent the transition to obesity in young adulthood. Results of the process evaluation will assess the barriers and enablers to widespread implementation of the text message program, with the ultimate goal of providing adolescents with age-appropriate, evidence-based, and accessible obesity prevention services.

The simple and innovative text message program addresses a critical gap in obesity prevention for adolescents, given their risk of future chronic diseases. Text messages are an appropriate and accessible intervention delivery modality for this population group. The benefit of text messages is that they do not require an internet connection to receive, offer an interactive modality to communicate with health professionals, can be delivered with minimal personnel, and may provide a socially equitable intervention for most adolescents. As such, text message interventions show the potential to reduce socioeconomic disparities in health care and deliver a scalable, low-cost intervention to a wide population. To date, there have been only eight RCTs investigating text messages for obesity prevention or management in adolescent populations [30,68-74]. The interventions were mostly multicomponent mobile health interventions, and there was limited process evaluation data to help understand the effects of the text message component. Taken together, currently, there is limited high-quality research investigating obesity prevention interventions delivered via text message for adolescents. Hence, the TEXTBITES study aims to address this research gap.

### Conclusion

This study will test the effectiveness of a six-month text message intervention to improve BMI and lifestyle outcomes for adolescents who are overweight and at risk of obesity. If effective at improving BMI and lifestyle outcomes, the results will provide evidence to inform future practice and community initiatives to promote obesity prevention behaviors for adolescents, and ultimately be translated to help all adolescents nationally and internationally.

## Abbreviations

ACAES: Australian Child and Adolescent Eating Survey
ASAQ: Adolescent Sedentary Activity Questionnaire
ANCOVA: analysis of covariance
BMI: body mass index
CESDR-10: Centre for Epidemiologic Studies Depression Scale-Revised
EDE-Q: Eating Disorder Examination questionnaire
PedsQL: Pediatric Quality of Life Inventory
PSQI-Short: Pittsburgh Sleep Quality Index Short
RCT: randomized controlled trial
SMS: short message service
TEXTMEDS: TEXT messages to improve MEDication adherence and Secondary prevention
TEXTBITES: TEXT message Behavioral Intervention for Teens on Eating, physical activity, and Social wellbeing
